# Palliative inpatients in general hospitals: a one day observational study in Belgium

**DOI:** 10.1186/1472-684X-10-2

**Published:** 2011-03-02

**Authors:** Marianne S Desmedt, Yolande L de la Kethulle, Myriam I Deveugele, Emmanuel A Keirse, Dominique J Paulus, Johan J Menten, Steven R Simoens, Paul J vanden Berghe, Claire M Beguin

**Affiliations:** 1 Unité de Soins Continus, Cliniques Universitaires Saint Luc, Université Catholique de Louvain, 10 avenue Hippocrate, 1200 Bruxelles, Belgique; 2 Centre Interdisciplinaire en Economie de la Santé, Ecole de Santé Publique, Université Catholique de Louvain, clos Chapelle aux Champs, 30, 1200 Bruxelles, Belgique; 3 Department of General Practice and Primary Health Care, Universiteit Gent, De Pintelaan 185, 9000 Gent, België; 4 Federatie Palliatieve Zorg Vlaanderen, Vander Vekenstraat 158, 1780 Wemmel, België; 5 Centre Fédéral d'Expertise des Soins de Santé, Centre Administratif du Botanique, Door Building (10° étage), Bd du Jardin Botanique 55, 1000 Bruxelles, Belgique; 6 Radiotherapie-oncologie, Universitair Ziekenhuis Leuven, Katholieke Universiteit Leuven, Herestraat 49, 3000 Leuven, België; 7 Onderzoekscentrum voor Farmaceutische Zorg en Farmaco-economie, Faculteit Farmaceutische Wetenschappen, Katholieke Universiteit Leuven, Herestraat 49, 3000 Leuven, België; 8 Informations et Statistiques Médicales, Cliniques Universitaires Saint Luc, Université Catholique de Louvain, 10 avenue Hippocrate, 1200 Bruxelles, Belgique

## Abstract

**Background:**

Hospital care plays a major role at the end-of-life. But little is known about the overall size and characteristics of the palliative inpatient population. The aim of our study was to analyse these aspects.

**Methods:**

We conducted a one-day observational study in 14 randomly selected Belgian hospitals. Patients who met the definition of palliative patients were identified as palliative. Then, information about their socio-demographic characteristics, diagnoses, prognosis, and care plan were recorded and analysed.

**Results:**

There were 2639 in-patients on the day of the study; 9.4% of them were identified as "palliative". The mean age of the group was 72 years. The primary diagnosis was cancer in 51% of patients and the estimated life expectancy was shorter than 3 months in 33% of patients and longer than 1 year in 28% of patients. The professional caregivers expected for most of the patients (73%), that the treatment would improve patient comfort rather than prolong life. Antibiotics, transfusions, treatments specific to the pathology, and artificial nutrition were administered in 90%, 78%, 57% and 50% of the patients, respectively, but were generally given with a view to controlling the symptoms.

**Conclusions:**

This analysis presents a first national estimate of the palliative inpatient population. Our results confirm that hospitals play a major role at the end-of-life, with one out of ten inpatients identified as a "palliative" patient. These data also demonstrate the complexity of the palliative population and the substantial diversity of care that they can require.

## Background

WHO defined palliative care as ''the active total care of patients whose disease is not responsive to curative treatment' [1]. In Belgium, palliative care relies on this definition and is provided in different settings: home care, residential units or hospices. These 51 residential units have 379 palliative beds, i.e. an insufficient number of beds in view of the palliative care demand. Therefore, every acute hospital is subsidized for an intramural palliative support team. Recently, 6 day centres have been created in Belgium. All these services are specialized in the care of palliative patients with more complex needs. The patients not treated in these services are treated in classic structures providing palliative care but not specialized in palliative care (general hospital wards, home care nursing services, general practitioners, nursing homes...) [2]. Beside the specialised palliative services, the country is divided into 25 areas, called palliative networks, each covering around 300.000 inhabitants. These networks coordinate the intervention of palliative care services and integrate them into the health care system. Each network has its own home care team.

Even though more than 70% of Belgian population would prefer to be treated at home, a large number of deaths occur in hospital [3]. A considerable amount of information regarding palliative inpatients is available and the place of death and its determinants in this specific context have been extensively investigated [4]. Van den Block et al. reported on the institutionalised nature of the final phase of life and concluded that clinical condition, expression of preferences, and characteristics of healthcare organisation seem to be associated with the transfer of such patients to the hospital [5]. However, the wider use of the hospital setting in providing palliative care has been less frequently considered. Most of the previous studies have been limited to specific diagnoses (e.g., cancer), age groups (e.g., the elderly), or settings (e.g., specialist palliative care services). Although some surveys have included the number of palliative inpatients, they do not give a global overview as they were all conducted in one single or in a university hospital [6-10]. Furthermore, two of them have focused only on terminally ill patients and another study was specifically interested in the need for specialist inpatient palliative care [6,7,10].

The purpose of this study was to assess the overall population of hospital palliative inpatients who should benefit from palliative care and to describe their main demographic and medical characteristics.

## Methods

The study was prospective and data was collected by interviews conducted with medical and nursing staff.

The literature shows that the proportion of cancer patients among palliative inpatients varies around 50% [6]. The sample size was computed to detect a 25% difference between the proportions of cancer and non cancer patients. With a hypothesis of type I error equal to 0.05 and a power equal to 0.80, the sample size should be 364 palliative patients. In order to estimate the number of beds to include, we chose a proportion of palliative patients of 10% as this proportion varies between 5% and 15% in the literature [6,9]. This would lead to investigate 364/0.10 = 3640 inpatients. Taking an occupation rate of 0.75 entails that 4842 beds should be checked.

Fourteen hospitals were randomly selected from all Belgian hospitals, taking into account the type (academic/non academic; acute/non acute), the size (< 300 beds, 300-500 beds and >500 beds), and the geographical location (Brussels, Flanders and Wallonia). Twelve categories were created based on these hospital's characteristics and each Belgian hospital was allocated to a single category. The hospital sample was built by a random selection of one hospital in each category. Finally, a university hospital from Flanders and another from Wallonia were added to the sample.

Two non university hospitals refused to participate and were replaced by 2 other hospitals randomly selected in the corresponding categories.

All hospital beds were included (4746 beds), except obstetrics and psychiatry as such wards are only rarely involved in palliative care. Intensive care units were also excluded although these units present a high death rate and in spite of innovative and specific palliative care programs [11]. Indeed the survey design was not adapted to this type of patients and treatments (prognostic uncertainties, specific life-sustaining treatment...). Paediatric and neonatology units were also not eligible due to their specific care plans. As we intended to measure the frequency of patients who could be considered as palliative and therefore should benefit from a palliative care program, the palliative units were not included in this survey. All patients hospitalised in an eligible unit for at least 48 hours (i.e. enough time for the physicians and the nurses to know the patient) were included in the study. Patients undergoing in-hospital transfer during the same stay were included only once.

The Ethics Committee of St Luc Hospital-UCL gave a positive opinion to the protocol which was registered in the National Federal Experimentation Data Bank of the National Ethics Committee under the number B40320084090. The study was performed according the different Belgian laws concerning confidentiality and private life (2002), patients' rights (2002) and experimentation on human beings (2004). This study was a sub-project of a larger one which was asked by the promoter, the Belgian Health Care Knowledge (KCE).

Three specifically appointed nurses conducted the survey in 2008, over a 3-month period. After hospital approval, the caregivers of each ward included in the survey were interviewed once on a certain day fixed in advance. In small hospitals with few wards, all interviews were conducted in a single day. Larger hospitals with a greater number of wards to be surveyed required more time. Only 3 physicians refused to participate, i.e. less than 1% of the physicians contacted.

The study nurses interviewed the nurses and physicians who were in charge of the patients and had daily contact with them. Firstly, the nurses and physicians were separately asked to assess whether that patient met the definition of a palliative patient.

In 2002, the World Health Organisation defined palliative care as "*an approach that improves the quality of life of patients and their families facing the problem associated with life-threatening illness through the prevention and relief of suffering by means of early identification, impeccable assessment and treatment of pain and other problems physical psychosocial and spiritual*" [12]. This definition is broader than the one given by the World Health Organisation in (1990): *"Palliative care is the active total care of patients whose disease is not responsive to curative treatment" *[1]. In Belgium, even though the notion of palliative patient is not officially defined, preferential reimbursement rate are granted to the patient having a 'palliative' status. As mentioned by Keirse et al. and by Pastrana et al., there is no current consensus about the definition of palliative patient [13,14]. Therefore within the context of our survey we used the following definition: "*a patient suffering from an incurable, progressive, life-threatening disease, with no possibility of obtaining remission, stabilization or improvement of this illness*". The term "*incurable*" excluded illnesses for which there is a chance of complete cure; the term "*progressive*" eliminated chronic, incurable but stable disease; the terms "*no possibility of obtaining remission, stabilization or improvement of the illness*" highlighted the ineffectiveness of specific therapeutics to control the disease [15]; and the term "l*ife-threatening disease*" introduced a notion of survival prediction and fatal outcome. This last notion could not be defined in a more precise way due to the difficulty in giving an accurate prognosis except when the patient is very close to death [16]. This definition of "palliative patient" does not include any criterion based on patient needs because we considered this aspect too difficult to define precisely, as it would have required taking into account many other factors deemed by the caregivers to be related to palliative care [17].

The second part of the study was carried out in respect of those patients who met these four criteria. The study nurses made an interview of the same caregivers using a structured questionnaire. The questionnaire collected data relating to the patient's socio-demographic characteristics, diagnoses, prognosis, and care plan. The questionnaire can be found in annex (File 1: English version; File 2: French version).

In order to avoid any (mis)interpretation of the palliative patient's definition, the term 'palliative' was not used during the interview of the caregivers. The interviewer presented to the caregivers a paper mentioning the 4 abovementioned criteria of our definition. Then the caregivers reviewed if the patient met each of the four criteria presented.

Before the beginning of the hospital survey, the questionnaire was first tested in 2 hospitals not included in the survey's sample. Then, the study nurses performed in one hospital under the supervision of the survey's designer. Finally, during the survey, regular meetings were organised between the study nurses and the survey's designer.

Univariate and multivariate statistics were performed to analyse the data. Pearson's chi square test was used to detect statistical differences between groups as the data were primarily categorical. Comparisons of age between groups were made using the Wilcoxon's test. Multivariate logistic regression was also used to test the effects of various factors on the intention to prolong life. The covariates introduced in the model were age, sex, pathology, prognosis and type of bed. The analysis was made with SAS version 9.1 and a p-value equal or less than 0.05 was considered as significant.

## Results

### Prevalence of palliative patients

Two-hundred and forty-nine in-patients were identified as "palliative" by the medical and nursing staff, comprising 9.4% of the total in-patient population. Table 1 shows that the prevalence of palliative patients was significantly less in surgical and rehabilitation beds than in medical and geriatric beds. The prevalence also varied significantly among regions. One Brussels' hospital had a particularly high prevalence value. After exclusion of this 'outlier', there were no significant differences in the prevalence of palliative patients between university and other hospitals, private or public institutions, and hospitals with or without a palliative care unit.

**Table 1:** Prevalence of palliative inpatients

		N	Palliative Patients	Non Palliative Patients	p-value
Type of beds	Surgery	727	16 (2.2%)	711(97.8%)	p ≤ 0,001
	
	Rehabilitation	488	22 (4.5%)	466 (95.5%)	
	
	Medicine	1015	134 (13.2%)	881 (86.8%)	
	
	Geriatric	409	77 (18.8%)	332 (81.2%)	

Region	Flanders	624	49 (7.9%)	575 (92.1%)	p ≤ 0,001
	
	Wallonia	692	56 (8.1%)	636 (91.9%)	
	
	Brussels	534	85 (15.9%)	449 (84.1%)	

Palliative care unit	With	1614	166 (10.3%)	1448 (89.7%)	p = 0.0610
	Without	1025	83 (8.1%)	942 (91.9%)	

Social status	Public	1111	96 (8.6%)	1005 (91.4%)	p = 0.2338
	
	Private	1528	153 (10.0%)	1375 (90.0%)	

### Patients' characteristics

Of the 249 palliative patients, 55% (136/249) were female and the mean age was 72 years with a range of 21 to 99. The majority of patients were older than 65 years (175/249) with a considerable number older than 80 (93/249). About half of the patients were married (112/239) and the others were widowed (79), divorced (14) or single (34). Seventy percent of patients (174/249) had been admitted from home and 30% from another residence, such as a nursing home.

**Figure 1:**
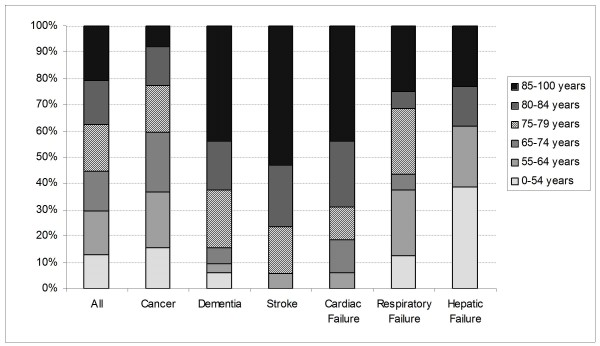
**Age distributions of palliative patients by diagnostic group**. The first bar represents the distribution of age of all palliative patients whatever the disease. The others display the distribution of age for the most frequent underlying diseases.

The primary diagnoses are shown in Table 2. Approximately half of the patients had a primary diagnosis of cancer. The most common non-cancer diagnoses were dementia, stroke, and cardiac, respiratory or hepatic failure. Cancer patients were significantly younger than the non-cancer patients (68 ± 14 versus 77 ± 13 years, p < 0.0001) and mainly hospitalised in medical beds (81/128). Patients suffering from dementia and stroke were significantly older than the other palliative patients (82 ± 9 years versus 70 ± 15 years, p < 0.0001) and were largely hospitalised in geriatric beds (34/49) (Figure 1).

**Table 2:** Distribution of primary diagnoses

Cancer diagnoses	128 (51.4%)
Solid tumour	108 (43.4%)

Haematological cancer	19 (7.6%)

Non-cancer diagnoses	121 (48.6%)

Dementia	32 (12.9%)

Stroke	17 (6.8%)

Organ system failure	50 (20.0%)

Cardiac failure	16 (6.4%)

Respiratory failure	16 (6.4%)

Hepatic failure	13 (5.2%)

Renal failure	5 (2.0%)

Other diseases	40 (16.0%)

Neurological diseases	8 (3.2%)

Vascular diseases	6 (2.4%)

Infectious diseases	2 (0.8%)

Others	6 (2.4%)

Total	249 (100%)

For almost one third of patients (71/242), the diagnosis had been established 3 months before the current hospitalisation and for half of them (112/242), it had been established during the year prior to the study.

**Figure 2:**
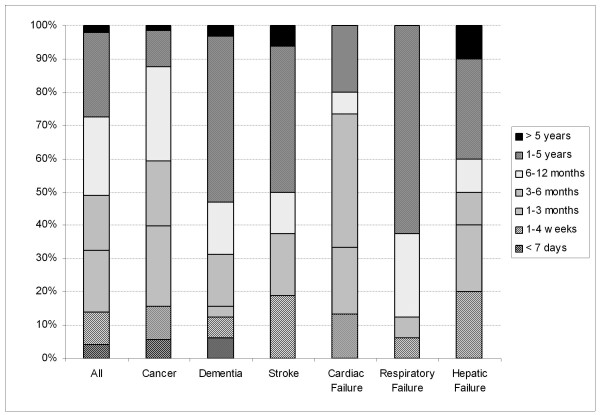
**Survival prognosis according to disease**. The first bar represents the distribution of life expectancy of all palliative patients whatever the disease. The others display the distribution of survival prognosis for the most frequent underlying diseases.

As illustrated in Table 3 the estimated life expectancy varied from less than 7 days to more than 5 years. One third of patients had a life expectancy of three months or less and, from the primary diagnosis, one half of patients would be expected to still be alive after six months (Figure 2). The prognosis was less than 1 year for 88% of cancer patients and for 56% of non-cancer patients (p < 0.0001).

**Table 3:** Distribution of estimated life expectancy

< 7 days	10 (4.1%)	79 (32.5%)
	
> 1 and ≤ 4 weeks	24 (9.9%)	
	
> 1 and ≤ 3 months	45 (18.5%)	
> 3 and ≤ 6 months	40 (16.5%)	97 (40.0%)
	
> 6 and ≤ 12 months	57 (23.5%)	

> 1 and ≤ 5 years	62 (25.5%)	67 (27.5%)
	
> 5 years	5 (2.0%)	

Total	243 (100%)	

### Patients' treatment

The caregivers had already established a treatment plan for 220 of the 249 patients (88%) before the interview. The plan had been discussed by physicians and nurses in 191 cases (77%) and by physicians alone in 27 cases (11%). These percentages were independent of the pathology, survival prognosis, or expectations of the treatment plan.

When analysing the expectations of the treatment plan, it appears that the goal of physicians and nurses was to improve patient comfort rather than prolonging life. Multivariate logistic regression analysis showed that treatment expectations depended mainly on the patient's prognosis (OR = 1.760, CL = 1.360 - 2.279). For the 79 patients with a survival prognosis less than 3 months, an expectation of prolonging life was reported in just 6.3% of cases (5/79), while for the 67 patients with a prognosis of at least 1 year, the expectation of prolonging life was reported in 41.8% of cases. No significant differences in expectations were observed comparing cancer to non-cancer patients (Table 4); however, the caregivers more frequently reported an expectation to prolong life in patients with stroke (6/17) or organ system failure (20/50) than in patients with dementia (1/32).

**Table 4:** Physicians' expectations from the treatment plan

		Life prolongation n = 65 (100%)	Improvement of comfort n = 179 (100%)	
	
Pathology	Cancer	32 (49.2%)	94 (52.5%)	p = 0.6501
		
	Non-cancer	33 (50.8%)	85 (47.5%)	
Age	< 75 years	37 (56.9%)	72 (40.2%)	p = 0.0204
		
	≥ 75 years	28 (43.1%)	107 (59.8%)	

Prognosis	≤ 3 months	5 (7.7%)	73 (40.8%)	p < 0.0001
		
	> 3 months	56 (86.2%)	104 (58.1%)	
		
	≤ 1 year	33 (50.8%)	141 (78.8%)	p = 0.0019
		
	> 1 year	28 (43.1%)	36 (20.1%)	

The type of treatment was generally clearly documented and defined by the professional caregivers. Cardiac resuscitation was excluded for 71% of patients (177/249). Antibiotics, transfusions, treatments specific to the causative pathology (e.g., chemotherapy for cancer patients), artificial nutrition, and transfer to the intensive care unit were being considered, had been planned, or were ongoing in 90% (224/249), 78% (195/249), 57% (142/249), 50% (124/249) and 33% (81/249) of patients, respectively. These interventions were administered in order to control symptoms in 66% (149/224), 74% (144/195), 56% (80/142), 45% (56/124) and 25% (20/81) of the cases, respectively. Table 5 shows the proportion of patients for which treatments were excluded depending on their prognosis. In case of very poor prognosis, cardiac resuscitation and transfer to intensive care unit were excluded for all patients. These 2 treatments were excluded for more than half of patients whatever their prognosis.

**Table 5:** Prognosis and treatment excluded

	Prognosis					
	
Treatments excluded	< 7 days	1-4 weeks	1-3 months	3-6 months	6-12 months	> 1 year
Cardiac resuscitation	100%	91.7%	81.8%	72.5%	63.2%	67.2%

Transfer to intensive care unit	100%	95.5%	72.7%	57.9%	58.2%	56.5%

Treatment specific to the causative disease	90.0%	52.2%	54.8%	30.8%	28.6%	33.3%

Artificial nutrition	90.0%	73.9%	47.6%	54.1%	40.0%	36.1%

Antibiotics	80.0%	30.4%	4.4%	0	1.8%	3.1%

Transfusion	80.0%	54.2%	20.5%	13.5%	9.1%	5.0%

## Discussion

Several limitations should be mentioned when interpreting these results. The first is a possible bias in the recruitment of patients. The initial sample size calculations gave an estimate of at least 3640 beds to recruit 364 palliative patients. The final sample size was smaller, i.e. 249 patients, for two reasons. The mean bed occupation rate was lower than expected; the study excluded patients with a length of stay less than 48 hours (25% of patients in some acute hospitals). The second limitation is related to the fact that only the health care providers were interviewed and therefore, the survey inaccurately reflects patient treatment preferences. The third limitation concerns the method of patient selection. We quantified the palliative in-patient population on just one day. Visiting the same hospitals more than once would have provided a more precise measure.

Nevertheless, this is the first survey to explore the number and characteristics of palliative inpatients at a national level. Slightly less than one out of ten inpatients was identified as palliative. Similar percentages have been reported from other surveys but these were conducted in just one institution. Morize et al included all patients with advanced or terminal stage life-threatening illness who were hospitalised in a large French university hospital [6]. These authors reported that 13% of the inpatients were palliative patients. A similar percentage (12%) was observed by Edmonds et al. and by Billings et al in an English and an American academic hospital, respectively [7,8]. In a study by Gott and colleagues, the prevalence of palliative patients was higher (22%), but the inclusion criteria in this study were based on need for supportive and palliative care [9]. Similarly, Skilbek et al. concentrated on specialist palliative care services and observed that 4% of in-patients were considered suitable for referral to a palliative care unit [10].

As expected, the largest numbers of palliative in-patients were admitted to geriatric beds. Metropolitan hospitals also had greater number of palliative patients, supporting the findings of Houtekkier et al. who concluded that palliative patients died in hospital more often in Brussels than in other parts of country [18].

As mentioned in the section 'method', one hospital was considered as an 'outlier'. This hospital holds 22% of Brussels' palliative beds and was the pioneer of in-hospital palliative care in the country. The higher proportion of palliative patients observed in this hospital could be due to a different culture about palliative care.

The second aim of the present study was to describe the demographic and clinical characteristics of the palliative inpatients. Our results show that the palliative inpatient population is complex. A large number of cancer and non-cancer pathologies are represented, contrary to what is usually described in specialist palliative care services [19,20]. As in previous reports, cancer was the leading diagnosis in our study but nearly half the patients had a non-malignant disease [6,9]. Despite their typically insidious onset, prolonged disease trajectory and difficulty in predicting life expectancy, chronic illnesses are major causes of death in developed countries today [21-23]. Moreover, persons dying from chronic illness tend to have frequent exacerbations requiring hospitalisation. There is evidence suggesting that these patients may require palliative care, just as those who suffer from malignant disease [24]. In view of these findings, palliative care in hospital cannot be confined to one patient group and professional caregivers need to acquire sufficient expertise to meet the common but also the specific needs of cancer and non-cancer patients [13].

Even if a correct estimation of the patient's prognosis remains quite difficult, our results show considerable variation in the life-expectancy of our palliative population [16]. One third of the palliative inpatients had a life expectancy of 3 months or less. These patients can be considered "terminally ill patients" as they are referred to in the current literature. For approximately another one third of patients, the physicians and nurses considered the survival prognosis to be more than one year.

Nevertheless, in 70% of all the palliative patients, the treatment plan aimed at improving symptoms rather than at prolonging life. As other researchers, we noted that when caregivers considered using potentially life-prolonging interventions, their decision was significantly associated with a long-term survival prognosis [25]. However, we found no difference between patients with and those without cancer, as reported by Van den Block et al [26]. In summary, in contrast to Becker and colleagues who noted that comfort-focused care concerned less than one dying patient out of two, our results indicate that the Belgian physicians and nurses are willing to limit aggressive treatments and to plan comprehensive palliative care [27]. Our results also indicate that the caregivers seem to be in agreement with the World Health Organisation's recommendation and tend to integrate palliative care as soon as possible into the course of illness, as reported by the majority of European medical oncologists [1,12,28].

Another striking finding of our study was that antibiotics, blood transfusions, specific treatments for primary disease, artificial nutrition, and transfer to the intensive care unit were considered for, or given, to about 90%, 80%, 60%, 50% and 33% of the patients, respectively. Several authors have already noted that in an acute care hospital, such therapeutic interventions, considered as comfort care, were continued for the majority of dying patients [26,29,30]. However, as these studies were retrospective charts reviews, the detailed reasons for the therapeutic procedures could not be clearly determined. In our survey, the treatment objective was generally clearly noted by the health care providers: transfusions and antibiotics were used largely to alleviate symptoms; whereas admission to the intensive care unit, artificial nutrition, and specific disease-related treatments were used more to prolong life. Undoubtedly, interventions such as antibiotics may contribute to a better management of distressing symptoms [31]. Furthermore, the long-term prognosis of some patients and uncertainty about the short-term prognosis in others may encourage a mixed management strategy and an interaction between a curative and palliative approach. Nevertheless, in a setting where priority is given to life-supporting and life prolonging activities, some of these interventions may be considered invasive and futile [32]. Van Leeuwen et al, who observed oncology multidisciplinary meetings discussing potentially life prolonging treatments, came to the conclusions that before making a decision, healthcare professionals should gather extensive information about gains that may be expected from an intervention [33]. If case of doubt about whether or not to start or continue treatment, the patient's wish could be a decisive consideration. Unfortunately, the purpose of our study was not to assess whether the decisions of the interviewed healthcare professionals were medically and ethically appropriate.

## Conclusions

The ability of health care providers to recognise the patient as "palliative" and their readiness to limit aggressive treatment and plan palliative care is essential to improve palliative care for hospitalised patients. The main result of this article is that physicians and nurses identified 10% of inpatients as "palliative" patients and that they adopted a comfort care plan in 70% of cases. Our results highlight that the palliative inpatient population is multifaceted and that therapeutic procedures are varied and complex. These findings may help in planning the organisation of palliative care in health care systems. Models of care that embrace all end-of-life paths and meet the common but also the specific needs of every disease must be developed. Medical and nursing staff also need to be educated and supported in provision of care so that palliative patients can live with comfort and dignity whatever their primary diagnosis or their life expectancy. Another challenge is to determine how many and which of the 10% of palliative in-patients should have access to specialised palliative care services. These issues are important and require careful consideration if high quality of care is to be provided to all patients at the end of life.

## Competing interests

The authors declare that they have no competing interests.

## Authors' contributions

MSD conceived of the study, participated in its design and coordination and helped to the draft of the manuscript. YLK participated to the data analysis. CMB participated in the acquisition of data, statistical analysis and drafted the manuscript. All authors read and approved the final manuscript.

## Pre-publication history

The pre-publication history for this paper can be accessed here:

http://www.biomedcentral.com/1472-684X/10/2/prepub

## Supplementary Material

Additional file 1 **Questionnaire (English version)**. This file contains the questionnaire used by the study nurses when interviewing the caregivers. Click here for file

Additional file 2 **Questionnaire (French version)**. This file contains the questionnaire used by the study nurses when interviewing the caregivers. Click here for file
