# Supplementary material for: Palliative inpatients in general hospitals: a one day observational study in Belgium
Source: BMC Palliat Care. 2011 Mar 2;10:2. doi: 10.1186/1472-684X-10-2 (PMC3052175; doi:10.1186/1472-684X-10-2)
Supplement: Additional file 1 — Questionnaire (English version). This file contains the questionnaire used by the study nurses when interviewing the caregivers. [file 1472-684X-10-2-S1.DOC]

1. Patient’s identification

###### Patient number (anonymous):

###### Patient identified as« palliative»

######  by the physician and the nurse (without common assent)

 by the physician and the nurse (after common assent)

 only by the physician

 only by the nurse

1. Hospital identification and type of beds

###### Hospital identification ____________

###### 2.1. The patient is hospitalised in a bed of

######  Acute care

 Internal Medicine  General internal medicine

 Cardiology

 Gastroenterology

 Geriatrics

 Haematology

 Neurology

 Nephrology

 Oncology

 Pneumology

 Other ____________________________

 Surgery  General surgery

 Cardio-thoracic surgery

 Digestive surgery

 Neurosurgery

 Orthopaedics

 Other

 Other _____________________________

 Non acute care

 Geriatrics

 Psychogeriatrics

 Locomotor rehabilitation

 Neurological rehabilitation

 Cardiac rehabilitation

 General rehabilitation

 Other ___________________________

1. Social and demographic data

###### 3.1. The patient’s age is ___ years

###### 3.2. The sex of the patient is  Male

 Female

###### 3.3. The patient is  Married or cohabitant

 Widow

 Divorced

 Single

###### 3.4. Before hospitalisation the patient was  At home:

######  Alone or with a minor

######  With one adult

######  Unknown

 Nursing Home

 Other:_______________________________

 Unknown

1. Information about the ‘palliative’ disease

###### 4.1. The main disease considered as the cause of the ‘palliative’ status is:

 Cancer  Solid

 Hematopoietic

 Terminal cardiac failure

 Cerebral infarction

 Other terminal vascular disease

 Terminal respiratory failure (COPD,…)

 Terminal hepatic failure (Cirrhosis,…)

 Terminal kidney failure

 Dementia

 Other neurological disease (Amyotrophic lateral sclerosis, Parkinson, Multiple sclerosis…)

 Incurable infectious disease (AIDS…)

 Other _______________________________

###### 4.2. The duration between the first diagnosis time of this disease is:

 ≤ 1 month

 > 1 and ≤ 3 months

 > 3 and ≤ 6 months

 > 6 and ≤ 12 months

 > 1 and ≤ 2 years

 > 2 and ≤ 5 years

 > 5 and ≤ 10 years

 > 10 years

###### 4.3. The estimated patient’s prognosis was:

######  < 7 days

 > 1 and ≤ 4 weeks

 > 1 and ≤ 3 months

 > 3 and ≤ 6 months

 > 6 and ≤ 12 months

 > 1 and ≤ 5 years

 > 5 years

1. Information about treatment plan

###### 5.1. What was the therapeutic whish of the different persons?

|  | To prolong life | Only to improve comfort | No wish expressed | I don’t know |  |
| --- | --- | --- | --- | --- | --- |
| Patient |  |  |  |  |  Not able to speak |
| Family |  |  |  |  |  absent or not able to speak |
| Physician |  |  |  |  |  |
| Nurses |  |  |  |  |  |

5.2. What was the type of treatment planned?

|  | Excluded | Considered | Planned | Given now | Undefined |
| --- | --- | --- | --- | --- | --- |
| Cardiac resuscitation |  |  | - | - |  |
| Transfer to intensive care unit |  |  |  | - |  |
| Treatment specific to the causative disease |  |  |  |  |  |
| Artificial nutrition |  |  |  |  |  |
| Antibiotics |  |  |  |  |  |
| Transfusion |  |  |  |  |  |

5.3. What was the objective of the treatment?

*(Only if the treatment was considered, planned or given*)

|  | To prolong life | Only to control symptom | Only as psychological support for the patient | Other | Without precise objective |
| --- | --- | --- | --- | --- | --- |
| Transfer to intensive care unit |  |  |  |  |  |
| Treatment specific to the causative disease |  |  |  |  |  |
| Artificial nutrition |  |  |  |  |  |
| Antibiotics |  |  |  |  |  |
| Transfusion |  |  |  |  |  |

###### 5.4. The therapeutic project  Has been discussed between multidisciplinary team

 Has been discussed only between physicians

 Has not been discussed

###### The therapeutic project  Has been formalised and written

 Has been orally transmitted to the team

 Has not been transmitted to the team
