# Supplementary material for: Palliative inpatients in general hospitals: a one day observational study in Belgium
Source: BMC Palliat Care. 2011 Mar 2;10:2. doi: 10.1186/1472-684X-10-2 (PMC3052175; doi:10.1186/1472-684X-10-2)
Supplement: Additional file 2 — Questionnaire (French version). This file contains the questionnaire used by the study nurses when interviewing the caregivers. [file 1472-684X-10-2-S2.DOC]

1. Identification du patient

###### Clé d’identification du patient ____________

###### 1.1. Patient identifié comme « palliatif »  Par le médecin et l’infirmier (sans recherche d’un consensus)

 Par le médecin et infirmier mais recherche d’un consensus

 Uniquement par le médecin

 Uniquement par l’infirmier

1. Identification de l’institution de soin et du type de lit hospitalier

###### Numéro d’identification de l’institution ____________

###### 2.1. Le patient est hospitalisé dans un lit de

######  Soins aigus (lit de court séjour)

 Médecine interne  Médecine interne générale (polyvalente)

 Cardiologie

 Gastro-entérologie

 Gériatrie

 Hématologie

 Neurologie

 Néphrologie

 Oncologie

 Pneumologie

 Autre ____________________________

 Chirurgie  Chirurgie générale

 Chirurgie cardio-thoracique

 Chirurgie digestive

 Neurochirurgie

 Chirurgie orthopédique

 Autre chirurgie

 Autre _____________________________

 Lit de moyen séjour

 Gériatrie

 Psycho-gériatrie

 Revalidation locomotrice

 Revalidation neurologique

 Revalidation cardiorespiratoire

 Revalidation « générale »

 Autre ___________________________

1. Données sociodémographiques concernant le patient

###### 3.1. Le patient est âgé ___ ans

###### 3.2. Le patient est de sexe  Masculin

 Féminin

###### 3.3. Le patient est  Marié ou cohabitant

 Veuf

 Divorcé ou séparé

 Célibataire

###### 3.4. Avant d’être hospitalisé le patient séjournait  Domicile

 Seul ou avec une personne mineure

 Avec au moins une personne majeure

 Je ne dispose pas de renseignement

 Maison de repos (et de soins)

 Autre lieu ___________________________

 Je ne dispose pas du renseignement

1. Données cliniques concernant la pathologie «palliative»

###### 4.1. Quelle est la nature de la pathologie principale justifiant le statut « palliatif »

 Cancer  Tumeur solide

 Hémo-lymphopathie

 Insuffisance cardiaque terminale

 Accident vasculaire cérébral

 Autre affection vasculaire terminale

 Insuffisance respiratoire terminale (BPCO,…)

 Insuffisance hépatique terminale (cirrhose,…)

 Insuffisance rénale terminale

 Démence

 Autre maladie neurologique dégénérative (SLA, PKS, SEP…)

 Maladie infectieuse incurable (SIDA…)

 Autre pathologie _______________________________

###### 4.2. Combien de temps s’est écoulé depuis le diagnostic initial de cette pathologie

 ≤ 1 mois

 > 1 et ≤ 3 mois

 > 3 et ≤ 6 mois

 > 6 et ≤ 12 mois

 > 1 et ≤ 2 ans

 > 2 et ≤ 5 ans

 > 5 et ≤ 10 ans

 > 10 ans

###### 4.3. Estimation du pronostic de vie

######  < 7 jours

 > 1 et ≤ 4 semaines

 > 1 et ≤ 3 mois

 > 3 et ≤ 6 mois

 > 6 et ≤ 12 mois

 > 1 et ≤ 5 ans

 > 5 ans

1. Description du projet thérapeutique

###### 5.1. Quel est le souhait des différents intervenants à l’égard du projet thérapeutique ?

|  | Prolonger la vie | Uniquement améliorer le confort | N’a exprimé aucun souhait particulier | Je ne dispose pas de ce renseignement |  |
| --- | --- | --- | --- | --- | --- |
| Patient |  |  |  |  |  incapable de s’exprimer |
| Proche(s) |  |  |  |  |  absent ou incapable de s’exprimer |
| Equipe médicale |  |  |  |  |  |
| Equipe infirmière |  |  |  |  |  |

5.2. Quelle est la nature et le type de soins et de traitements qu’il est prévu d’administrer ?

|  | Exclu | Envisageable | Programmé | En cours | Non défini |
| --- | --- | --- | --- | --- | --- |
| Réanimation cardiorespiratoire |  |  | - | - |  |
| Admission en unité de soins intensifs |  |  |  | - |  |
| Traitement « actif » de la pathologie « palliative » |  |  |  |  |  |
| Alimentation artificielle (entérale ou parentérale) |  |  |  |  |  |
| Antibiothérapie |  |  |  |  |  |
| Transfusion d’un dérivé sanguin |  |  |  |  |  |

5.3. Avec quel objectif ces traitements sont-ils administrés ?

(Répondre uniquement si le traitement est envisageable, programmé ou en cours)

|  | Prolonger la vie | Uniquement contrôler  un symptôme | Uniquement soutenir psychologiquement le malade | Avec un autre objectif | Sans objectif précis |
| --- | --- | --- | --- | --- | --- |
| Admission en unité de soins intensifs |  |  |  |  |  |
| Traitement « actif » de la pathologie « palliative » |  |  |  |  |  |
| Alimentation artificielle (entérale ou parentérale) |  |  |  |  |  |
| Antibiothérapie |  |  |  |  |  |
| Transfusion d’un dérivé sanguin |  |  |  |  |  |

###### 5.4. Le projet thérapeutique  a été discuté en équipe pluridisciplinaire

 a été discuté uniquement au sein de l’équipe médicale

 n’a pas été discuté

###### Le projet thérapeutique  est formalisé par écrit

 a été transmis oralement à l’ensemble de l’équipe

 n’a pas été communiqué à l’ensemble de l’équipe
